# Strong and graded associations between level of asthma severity and all-cause hospital care use and costs in the UK

**DOI:** 10.1136/bmjresp-2023-002003

**Published:** 2023-12-14

**Authors:** Anya Jacobs, Runguo Wu, Florian Tomini, Anna De Simoni, Borislava Mihaylova

**Affiliations:** 1 Health Economics and Policy Research Unit, Wolfson Institute of Population Health, Queen Mary University of London, London, UK; 2 Asthma UK Centre for Applied Research, Wolfson Institute of Population Health, Queen Mary University of London, London, UK; 3 Centre for Primary Care, Wolfson Institute of Population Health, Queen Mary University of London, London, UK; 4 Nuffield Department of Population Health, University of Oxford, Oxford, UK

**Keywords:** Asthma, Health Economist

## Abstract

**Background:**

Hospital admissions account for a large share of the healthcare costs incurred by people with asthma. We assessed the hospital care use and costs associated with asthma severity using the UK Biobank cohort and linked healthcare data.

**Methods:**

Adult participants with asthma at recruitment were classified using their prescription data into mild and moderate-to-severe asthma and matched separately to asthma-free controls by age, sex, ethnicity and location. The associations of asthma, by severity, with the annual number of all-cause hospital admissions, days spent in hospital and hospital costs were estimated over a 10-year follow-up period using three specifications of negative binomial regression models that differed according to the sociodemographic and clinical characteristics adjusted for.

**Results:**

Of the 25 031 participants with active asthma, 80% had mild asthma and 20% had moderate-to-severe asthma. Compared with participants with mild asthma, those with moderate-to-severe asthma were on average 2.7 years older, more likely to be current (13.7% vs 10.4%) or previous (40.2% vs 35.2%) smokers, to have a higher body mass index (BMI), and to be suffering from a variety of comorbid diseases. Following adjustments for age, sex, ethnicity and location, people with mild asthma experienced on average 36% more admissions (95% CI 28% to 40%), 43% more days in hospital (95% CI 35% to 51%) and 36% higher hospital costs (95% CI 31% to 41%) annually than asthma-free individuals, while people with moderate-to-severe asthma experienced excesses of 93% (95% CI 81% to 107%), 142% (95% CI 124% to 162%) and 98% (95% CI 88% to 108%), respectively. Further adjustments for socioeconomic deprivation, smoking status, BMI and comorbidities resulted in smaller though still highly significant positive associations, graded by severity, between asthma and hospital use and costs.

**Conclusions:**

Strong graded associations are reported between asthma severity and the extent of hospital use and costs in the UK. These findings could inform future assessments of the value of asthma management interventions.

**WHAT IS ALREADY KNOWN ON THIS TOPIC**

- Asthma is a chronic disease affecting 12% of the UK population and represents a major burden to healthcare resources.
- Hospital admissions account for a large portion of the direct costs incurred by asthma but the scale of this burden is unclear in relation to disease severity.

**WHAT THIS STUDY ADDS**

- There are large differences in all-cause hospital use among the asthma population. While patients with mild asthma incurred about a third more hospital admissions and costs than matched asthma-free peers, patients with more severe asthma incurred on average twice more admissions and costs. Large differences persisted following adjustments for a range of sociodemographic, lifestyle and clinical characteristics.

**HOW THIS STUDY MIGHT AFFECT RESEARCH, PRACTICE OR POLICY**

- Significant cost savings can be made through the early detection of asthma patients at risk of progressing to more severe forms of the disease and targeting preventive therapies to these at-risk populations.
- Future assessments need to prioritise therapies to patients likely to benefit most according to their unique health profile and personal characteristics, enabling more stratified treatment decisions to be made within the healthcare resources available.

## Introduction

While asthma imposes a high burden internationally, its prevalence in the UK significantly exceeds the global average.^1 2^ 5.4 million people are currently receiving treatment for asthma in the UK, 4.3 million of whom are adults.^3^ Asthma is conservatively estimated to cost the UK public sector at least £1.1 billion every year.^4^ Nationwide, asthma is responsible for over 6 million primary care consultations, 100 000 hospital admissions^5^ and the loss of 17 million working days annually.^6^ The disease burden of asthma is unevenly distributed across the asthma population, with individuals suffering from more severe forms of disease experiencing greater morbidity and lower quality of life, accounting for a greater relative share of resources and costs.^7^

While there is no universal standardised method for defining asthma severity, there is a general consensus that more severe disease requires higher-intensity treatment,^8^ and thus asthma severity is most commonly determined according to the intensity of treatment required to control the disease.^9^ Such severity classifications are commonly used to target specific interventions to categories of asthma patients likely to benefit most efficiently.^10^ Therefore, there is an urgent need to estimate the impact of asthma severity on healthcare usage to inform such efforts and optimise the allocation of healthcare resources to the asthma population.

The great majority of healthcare costs are disproportionately consumed by patients with more severe forms of asthma, with hospitalisation usually occurring when asthma management fails to prevent an acute severe attack, a costly event to treat.^11^ Hospital admissions account for a significant portion of the healthcare costs incurred by asthma, representing approximately 9%–12% of the total National Health Service (NHS) expenditure for asthma.^5 12^ There is a notable lack of research investigating hospital costs specifically in relation to asthma severity status. This study aims to address this gap by providing a real-world evaluation of the hospital care use and hospital costs of UK adults with asthma using a population drawn from the UK Biobank study.^13^ By assessing the association of asthma severity with hospital care use, the study could enable more stratified treatment decisions such that therapies are targeted to the patients likely to benefit most according to their unique health profile.

## Methods

### Data source

This observational study used data from the UK Biobank database, a large ongoing cohort study of 502 540 participants aged 40–70 years at recruitment between 2006 and 2010 across the UK.^13^ ^14^ The study population includes individuals with asthma at entry into UK Biobank and matched asthma-free controls.

### Study population

Participants with asthma were required to have doctor-diagnosed asthma and take asthma medication as per the British Thoracic Society (BTS) steps 1–5^15^ at recruitment into UK Biobank (ie, had active asthma). Participants with doctor-diagnosed asthma were identified using the predefined asthma algorithm adopted by the UK Biobank.^16^ Participants were categorised by asthma severity according to the type and dosage of asthma medications they were taking at recruitment. Moderate-to-severe asthma was defined by BTS stages 3–5 criteria, as specified in Shrine *et al*.^17^ Participants taking medication from both mild and moderate-to-severe categories were categorised into the moderate-to-severe population.

Participants free from asthma at entry into UK Biobank were randomly selected to act as controls to participants with asthma. For each individual with asthma, 5 asthma-free controls matched for age, sex, ethnicity and geographical location (defined using the ‘assessment centre’ variable within UK Biobank) were randomly selected without replacement. Matching was conducted separately for mild and moderate-to-severe asthma patients, producing two asthma-free control cohorts. To identify controls, 5:1 nearest neighbour matching was performed with the Mahalanobis distance and without replacement using R’s ‘MatchIt’ package.^18^ The quality of matching was confirmed by assessing the balance of matched covariates by computing the standardised mean differences using R’s ‘cobalt’ package.^19^

### Hospital care outcomes

Linked Hospital Episode Statistics data are available for all hospital admissions of UK Biobank participants. Hospital episodes were mapped into Healthcare Resource Groups as part of an earlier study, with the cost of each hospital admission equalling the sum of the costs of all overlapping episodes.^20^ Hospital admissions were costed using the 2018/2019 national schedule of reference costs.^21^

The number of all-cause hospital admissions, days spent in hospital and costs of hospital care were established for each year of follow-up for each participant, from entry into UK Biobank to the point of censoring or death. Day hospital admissions, that is, patients admitted and discharged from hospital in the same day, were counted as half days in hospital. Cut-off dates for data collection were the earliest of the following: censoring dates for linked hospital data (29 February 2020 for England; 30 September 2016 for Scotland and 31 March 2016 for Wales), 29 February 2020 or death. The study follow-up period was further limited to follow-up years with less than 25% of censored participants. Participant follow-up years with incomplete information due to censoring were excluded, while years when deaths occurred were retained under the assumption that participants were fully observed prior to death.

### Statistical analysis

Annual hospital outcomes (number of admissions, days in hospital and hospital costs) were compared between mild and, separately, moderate-to-severe asthma, with asthma-free controls using negative binomial regression models. SEs, clustered by participant, were estimated using R’s ‘lmtest’ and ‘sandwich’ packages.^22 23^ Incident rate ratios, with 95% CIs, are reported. Three levels of covariate adjustments were prespecified: (1) minimally adjusted models with adjustments for the matching factors (age, sex, ethnicity and location) and calendar year; (2) intermediately adjusted models with adjustments for quintile of socioeconomic deprivation (as measured by the Townsend Deprivation Index) in addition to the matching factors and calendar year and (3) fully adjusted models with adjustments for smoking status, body mass index (BMI) category and comorbidities in addition to socioeconomic deprivation, matching factors and calendar year. Further analyses assessed the association of asthma with different types of hospital admission and whether associations between asthma severity and hospital outcomes differed with duration of follow-up or across quintiles of socioeconomic deprivation. Further details of the study methodology are available in online supplemental material 1. Analyses were executed using R software V.4.2.2. The code used in our analyses are available at https://github.com/anyajacobs/UKBiobank. An accompanying Excel calculator (online supplemental material 2) is available to illustrate the use of the minimally adjusted hospital cost models.

10.1136/bmjresp-2023-002003.supp1 Supplementary data

10.1136/bmjresp-2023-002003.supp2 Supplementary data

### Patient and public involvement

Preliminary results from this study were presented to and discussed with asthma patients and respiratory-focused health professionals within the Asthma UK Centre for Applied Research group. Patients endorsed the study design as enabling comparability by age, sex, deprivation and location, and the exploration of a wide range of characteristics (sociodemographic and comorbidities) associated with asthma.

## Results

**Figure 1:**
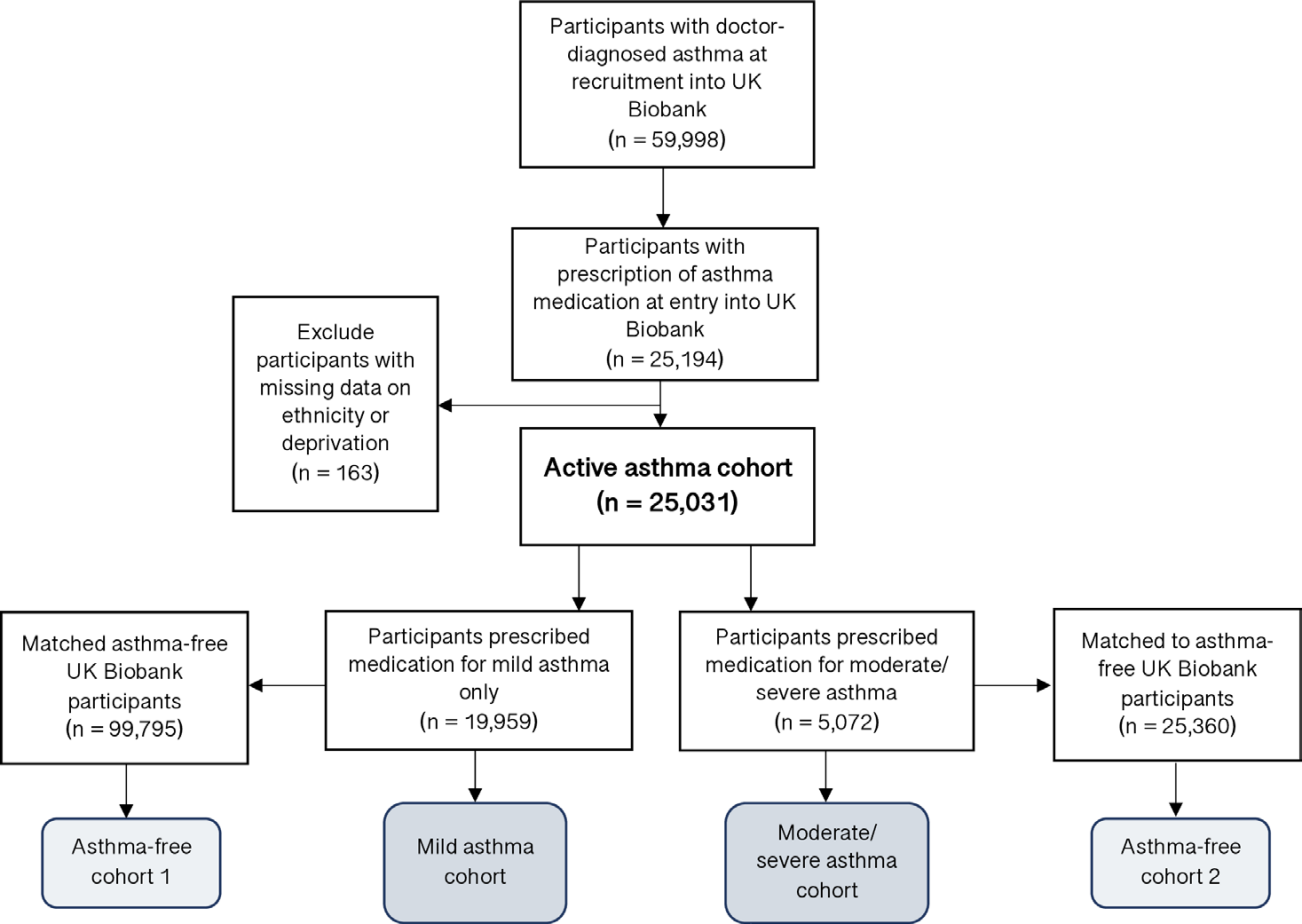
Study population.

A total of 25 194 participants with active asthma at recruitment were identified in UK Biobank. A total of 163 of these participants were excluded due to missing data on ethnicity and/or deprivation. The study population includes 25 031 participants with asthma (19 959 with mild asthma (80%) and 5072 with moderate-to-severe asthma (20%)) who were matched to separate asthma-free control cohorts (figure 1).

### Baseline characteristics of study participants

Compared with participants with mild asthma, those with moderate-to-severe asthma were on average 2.7 years older (table 1). In both cohorts, about 40% of participants were male. 95% and 96% of the mild and moderate-to-severe asthma cohorts were of white ethnicity, respectively.

**Table 1:** Baseline characteristics of study participants

Characteristic	Mild asthma (n=19 959)		Matched asthma-free cohort 1 (n=99 795)		Moderate-to-severe asthma (n=5072)		Matched asthma-free cohort 2 (n=25 360)	
	N (% or SD)		N (% or SD)		N (% or SD)		N (% or SD)	
Sex								
Male	8017	(40.2)	40 085	(40.2)	2036	(40.1)	10 180	(40.1)
Female	11 942	(59.8)	59 710	(59.8)	3036	(59.9)	15 180	(59.9)
Age, years								
Mean (SD)	55.94	(8.3)	55.94	(8.3)	58.66	(7.7)	58.66	(7.7)
<45	2395	(12.0)	11 968	(12.0)	319	(6.3)	1595	(6.3)
45–49	2903	(14.5)	14 522	(14.6)	480	(9.5)	2400	(9.5)
50–54	3110	(15.6)	15 550	(15.6)	628	(12.4)	3142	(12.4)
55–59	3469	(17.4)	17 346	(17.4)	908	(17.9)	4538	(17.9)
60–64	4455	(22.3)	22 274	(22.3)	1304	(25.7)	6522	(25.7)
≥65	3627	(18.2)	18 135	(18.2)	1433	(28.3)	7163	(28.2)
Ethnicity								
Black	322	(1.6)	1610	(1.6)	44	(0.9)	220	(0.9)
South Asian	340	(1.7)	1700	(1.7)	74	(1.5)	370	(1.5)
White	18 871	(94.5)	94 355	(94.5)	4871	(96.0)	24 355	(96.0)
Other	426	(2.1)	2130	(2.1)	83	(1.6)	415	(1.6)
BMI category, kg/m^2^		***				***		
<18.5	105	(0.5)	511	(0.5)	31	(0.6)	146	(0.6)
18.5–25	5566	(28.0)	33 710	(34.0)	1281	(25.4)	8101	(31.1)
25–30	7940	(40.0)	41 233	(41.5)	1914	(38.0)	10 792	(42.8)
30–35	4051	(20.4)	17 158	(17.3)	1136	(22.5)	4480	(17.7)
35–40	1422	(7.2)	4902	(4.9)	436	(8.7)	1248	(4.9)
40+	772	(3.9)	1777	(1.8)	241	(4.8)	473	(1.9)
Missing	153	(0.8)	505	(0.5)	33	(0.7)	120	(0.5)
Townsend deprivation score quintile		***				***		
1 (least deprived)	6923	(34.7)	38 238	(38.3)	1550	(30.6)	9964	(39.3)
2	3753	(18.8)	20 336	(20.4)	925	(18.2)	5225	(20.6)
3	3277	(16.4)	15 956	(16.0)	806	(15.9)	3974	(15.7)
4	3079	(15.4)	14 133	(14.2)	812	(16.0)	3414	(13.5)
5 (most deprived)	2927	(14.7)	11 132	(11.2)	979	(19.3)	2783	(11.0)
Smoking status		***				***		
Never smoked	10 791	(54.4)	55 529	(55.8)	2319	(46.1)	13 668	(54.1)
Current smoker	2070	(10.4)	10 407	(10.5)	689	(13.7)	2463	(9.8)
Previous smoker	6988	(35.2)	33 519	(33.7)	2025	(40.2)	9114	(36.1)
Missing	110	(0.6)	340	(0.3)	39	(0.8)	115	(0.5)
Comorbidity								
COPD	1154	(5.8)***	1261	(1.3)	1003	(19.8)***	370	(1.5)
Diabetes	1224	(6.1)***	4743	(4.8)	448	(8.8)***	1386	(5.5)
Hypertension	6220	(31.2)***	26 105	(26.2)	1996	(39.4)***	7682	(30.3)
Myocardial Infarction	522	(2.6)***	1994	(2.0)	255	(5.0)***	734	(2.9)
Stroke	397	(2.0)***	1524	(1.5)	168	(3.3)***	479	(1.9)
Cancer	1571	(7.9)	7695	(7.7)	525	(10.4)***	2191	(8.6)
Obstructive sleep apnoea	100	(0.5)***	296	(0.3)	42	(0.8)***	83	(0.3)
Chronic kidney disease	275	(1.4)*	1175	(1.2)	110	(2.2)***	342	(1.3)
Peripheral arterial disease	495	(2.5)***	2083	(2.1)	234	(4.6)***	639	(2.5)
Mental health disorder	2003	(10.0)***	7304	(7.3)	589	(11.6)***	1835	(7.2)
Duration of follow-up (years), mean (SD)	10.72	(1.36)	10.73	(1.33)	10.58	(1.58)	10.73	(1.37)

Control participants were matched to participants with mild or moderate-to-severe asthma, respectively, by age, sex, ethnicity and UK Biobank recruitment centre. χ^2^ tests were used to test for heterogeneity; p values are shown for comparisons of characteristics not used in matching between participants with asthma and respective controls.

Statistical significance: *p<0.05; **p<0.01; ***p<0.001.

BMI, body mass index; COPD, chronic obstructive pulmonary disease.

Higher levels of obesity (BMI ≥30 kg/m^2^) were noted among participants with asthma compared with their controls (31.5% vs 24% for mild asthma and 36% vs 24.5% for moderate-to-severe asthma, respectively (p<0.001)). While proportions of current and previous smokers were similar between participants with mild asthma and their controls, adults with moderate-to-severe asthma were more likely to be current (13.7% vs 9.8%) or previous (40.2% vs 36.1%) smokers compared with asthma-free controls (p<0.001). Greater proportions of participants with asthma were categorised into higher deprivation quintiles compared with their controls: 15.4% vs 14.2% in quintile 4 and 14.7% vs 11.2% in the most deprived quintile 5 for mild asthma, and 16% vs 13.5% in quintile 4 and 19.3% vs 11% in quintile 5 for moderate-to-severe asthma (p<0.001).

Higher prevalence of all comorbidities was observed among participants with asthma compared with their asthma-free controls. The mild asthma population had statistically significantly higher rates for all comorbidities, except cancer, relative to controls. They were more likely to have chronic obstructive pulmonary disease (COPD) (5.8% vs 1.3%), diabetes (6.1% vs 4.8%), hypertension (31.2% vs 26.2%), myocardial infarction (2.6% vs 2%), stroke (2% vs 1.5%), obstructive sleep apnoea (0.5% vs 0.3%), peripheral arterial disease (2.5% vs 2.1%), a mental health disorder (10% vs 7.3%) (all p<0.001) and chronic kidney disease (CKD) (1.4% vs 1.2%) (p<0.05). Compared with matched asthma-free controls, patients with moderate-to-severe asthma were more likely to have COPD (19.8% vs 1.5%), diabetes (8.8% vs 5.5%), hypertension (39.4% vs 30.3%), myocardial infarction (5% vs 2.9%), stroke (3.3% vs 1.9%), cancer (10.4% vs 8.6%), obstructive sleep apnoea (0.8% vs 0.3%), CKD (2.2% vs 1.3%), peripheral arterial disease (4.6% vs 2.5%) or a mental health disorder (11.6% vs 7.2%) (all p<0.001).

### Hospital outcomes

**Figure 2:**
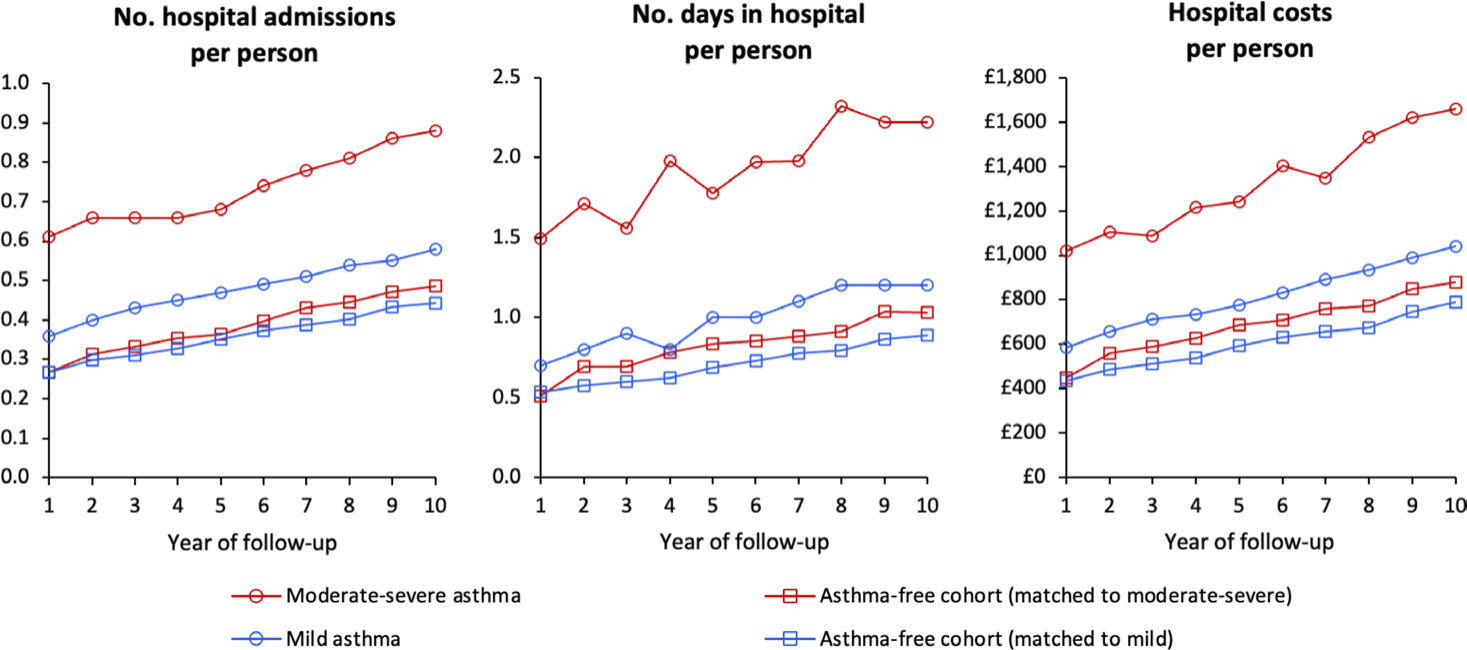
Annual per person hospital admissions, days spent in hospital, and hospital costs, by year of follow-up, for participants with mild asthma, moderate-severe asthma and their matched controls.

Descriptive analyses indicated that hospital admissions, days in hospital and hospital costs increased annually for all cohorts (online supplemental table S1). However, people with asthma consistently experienced more hospital admissions and spent more days in hospital, with greater associated costs, than their matched asthma-free counterparts, with increases being larger for those with moderate-to-severe asthma (figure 2).

Over the 10-year observation period, participants with mild asthma experienced 0.47 hospital admissions annually (vs 0.36 for controls), 0.99 days in hospital (vs 0.70) and annual hospital admission costs of £805 (vs £589) (all p<0.001; Online supplemental table S2). The corresponding annual statistics for patients with moderate-to-severe asthma were on average 0.72 annual hospital admissions, 1.90 days in hospital and hospital admissions costs of £1305 per participant (vs 0.38, 0.81 and £679 for matched controls, respectively). Diseases of the respiratory system (International Classification of Diseases, Tenth Revision (ICD-10 chapter) X, codes J00–J99)^24^ accounted for a greater proportion of hospital admissions in mild (6.3%) and moderate-to-severe (12.5%) asthma patients compared with their matched asthma-free controls (2.6% and 2.7%, respectively) over the 10-year study period (online supplemental table S3).

### Impact of asthma on all-cause hospital admissions, length of hospital stay and hospital costs

The estimated negative binomial regression models for annual hospital admissions, days spent in hospital and hospital costs over the first 10 years of follow-up post-recruitment into UK Biobank are reported in online supplemental tables S4–S9.

**Figure 3:**
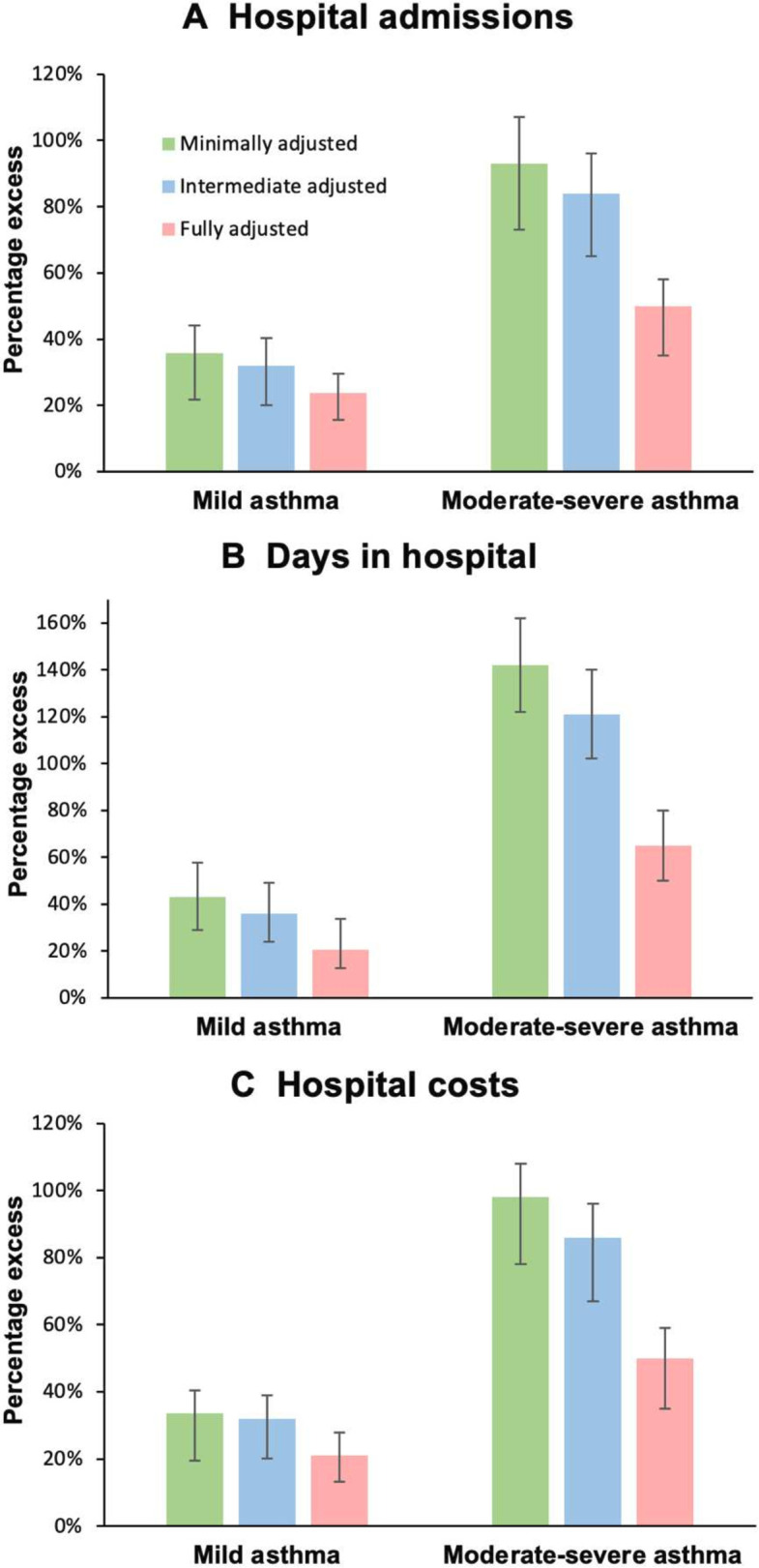
Excess annual hospital care use and costs (%) of people with asthma compared to controls. Excess annual hospital admissions, days spent in hospital and hospital costs experienced by individuals with mild and moderate-severe asthma compared to asthma-free controls (matched by age, sex, ethnicity, and location). All models included adjustments for calendar year and the matching factors. Minimally adjusted models (green) included no further adjustments. Intermediate adjusted models (blue) adjusted further for Townsend quintiles of socioeconomic deprivation in addition to the matching factors and calendar year. Fully adjusted models (red) adjusted further for smoking status, BMI category and comorbidities in addition to socioeconomic deprivation, matching covariates and calendar year.

The regression models across the three levels of adjustments confirmed strong associations of having asthma with more hospital admissions, spending more days in hospital and greater hospital costs than those without asthma (figure 3, online supplemental tables S4–S9). The extent of this excess varies largely according to disease severity. Compared with matched asthma-free controls, people with moderate-to-severe asthma experienced about three times greater increases in hospital admissions, time in hospital and incurred greater hospital costs than those with mild asthma. Following matching and adjustments for age, sex, ethnicity and geographical location, people with mild asthma on average experienced a 36% greater annual rate of hospital admissions (95% CI 28% to 40%), 43% more days in hospital (95% CI 35% to 51%) and 36% higher hospital costs annually (95% CI 31% to 41%) than asthma-free individuals, while people with moderate-to-severe asthma experienced corresponding excesses of 93% more admissions (95% CI 81% to 107%), 142% more days in hospital (95% CI 124% to 162%) and incurred 98% higher costs (95% CI 88% to 108%) relative to matched controls (online supplemental tables S4 and S7).

Adjusting further for quintile of socioeconomic deprivation reduced these associations (figure 3), reflecting the association between higher socioeconomic deprivation and higher asthma prevalence. People living in more deprived Townsend quintiles accounted for greater proportions of the excess hospital outcomes (online supplemental tables S5 and S8). Controlling for deprivation also attenuated the impact of ethnicity on hospital outcomes.

Adjusting for further patient characteristics, including smoking status, BMI and a range of comorbidities, resulted in further reduced though still highly significant positive associations between asthma and rates of hospital outcomes (all p<0.001) (online supplemental tables S6 and S9). For example, adjusting for these extra characteristics reduced the estimated associations of asthma with hospital costs from 32% to 21% (95% CI 17% to 25%) for mild and from 86% to 50% (95% CI 43% to 59%) for moderate-to-severe asthma, respectively, compared with intermediately adjusted models that exclude these as adjustments.

Testing for annual trend showed that hospital outcomes trended upwards across follow-up years for all cohorts, increasing annually between 6% and 8% according to model adjustments (online supplemental table 10), p<0.001). However, there was no evidence that these trends varied between participants with asthma and asthma-free controls.

### Impact of asthma across quintiles of socioeconomic deprivation

The relationship between asthma and socioeconomic deprivation was further explored in regressions that included interaction terms between deprivation quintiles and asthma (online supplemental table S11). The associations of asthma with hospital outcomes were stronger with higher levels of deprivation with evidence for linear trends across deprivation quintiles, which weakens with level of adjustments. In minimally adjusted models, mild asthma increases the rate of admissions, days in hospital and hospital costs by 6%, 5% and 4% per rise in quintile, respectively, while moderate-to-severe asthma increased these outcomes by 7%, 5% and 6%, respectively.

### Impact of asthma by category of hospital admission

The rates of hospital admissions according to their primary diagnosis were compared for people with and without asthma (online supplemental table S12). Asthma was associated with increased admissions rates across all diagnostic categories, with the greatest impact observed for respiratory-related hospital admissions (admissions assigned primary diagnoses from ICD-10 chapter X). Individuals with mild asthma experienced a rate of 3.3, 3.1 and 2.7 times as many respiratory hospital admissions compared with those without asthma in minimally adjusted, intermediately adjusted, and fully adjusted models, respectively. People with moderate-to-severe asthma experienced 10.5, 9.5 and 6.2 times as many respiratory hospital admissions compared with controls across the respective models.

### Impact of other participant characteristics on hospital admissions, days in hospital and costs

Being older at recruitment into UK Biobank, being male and being of any ethnicity other than white were associated with higher rates of hospital outcomes (online supplemental tables S4 and S7). Older age increased the rate of all hospital outcomes by 4%–5% per extra year of age, while being male had a particularly high association with the length of hospital stay (p<0.001). Smoking status had a pronounced impact, with current smokers having on average higher rates of hospital admissions, days in hospital, and costs by 27%, 57% and 38% (mild cohort, online supplemental table S6) and 33%, 59% and 45% (moderate-to-severe, online supplemental table S9) compared with those who had never smoked, respectively (p<0.001). Being a previous smoker was also associated with higher rates but to a much lesser extent. Having a BMI in any category outside of the healthy range of 18.5–25 increased all hospital outcomes, with the most extreme category; severely obese (BMI 40+), having the largest positive impact on rates (p<0.001). Various comorbidities were also significantly associated with excess hospital use and costs, with cancer and CKD having the greatest impact (p<0.001), followed closely by COPD (online supplemental tables S6 and S9). Regarding hospital costs specifically, the presence of cancer had the greatest impact compared with other diseases (p<0.001).

## Discussion

This study reports that adults with asthma experience on average more hospital admissions, more days in hospital and incur greater hospital costs than those without asthma, the extent of which varies significantly according to disease severity. Following matching and adjustments for age, sex, ethnicity and geographical location, people with mild asthma on average experienced 36% more admissions, 43% more days in hospital and 36% higher hospital costs than asthma-free individuals, while people with moderate-to-severe asthma experienced excesses of 93% more admissions, 142% more days in hospital and incur 98% more costs compared with asthma-free individuals. Further adjustments for socioeconomic deprivation, smoking status, BMI and a range of comorbidities led to reduced but still highly significant positive associations between asthma and rates of hospital outcomes. Having active asthma also had a profound impact on the rate of respiratory-related hospital admissions. Even in fully adjusted models, people with mild and moderate-to-severe asthma were estimated to experience a rate of 2.7 and 6.2 times more respiratory hospital admissions compared with those without asthma, respectively. Our results show that asthma burdens secondary care with significantly higher rates of hospital admissions, length of stay and hospital costs compared with the asthma-free population, with more severe disease contributing disproportionately to this burden, mirroring the findings of other studies within the asthma literature.^25–28^ It is likely, however, that the fully adjusted models in this study underestimate the full effect of asthma on hospital outcomes, since socioeconomic deprivation, smoking, BMI and the comorbidities included here are highly associated with asthma.^29^ We, therefore, recommend the minimally adjusted regressions as the preferred models for drawing overall associations between asthma and hospital resource use and costs. However, some of these overall associations are due to co-occurring comorbidities. A recent study using Canadian health administrative data and a similar matched cohort study design, found that severe asthma patients generated on average three times higher and two times higher annual healthcare costs compared with the general non-asthma population (US$4125 vs US$1345) and the non-severe asthma population (US$4125 vs US$2202), respectively.^30^ Over half of the difference in all-cause healthcare costs between severe and non-severe asthma were attributable to comorbidities, with a third of these costs originating from comorbidity-related hospitalisations. The most expensive comorbidity was respiratory conditions other than asthma, followed by diseases of the nervous and digestive systems.^30^ Similarly, our adjustments for socioeconomic deprivation, smoking, BMI and comorbidities reduced hospital admissions and costs associated with asthma by a third in mild asthma and by quarter to a third in moderate-to-severe asthma. Adults with asthma carry a significant burden of comorbid disease, particularly the moderate-to-severe population, which is likely to contribute to the increased healthcare consumption and costs we observed relative to those who are asthma-free, thus accounting for part of the association between asthma and hospital outcomes. These findings highlight the importance of considering the burden of multimorbidity in evidence-informed decision making for asthma.

Our findings identify several patient subgroups at greater risk of hospitalisation, including those who were current smokers, overweight or suffered from comorbid diseases such as COPD, cancer and CKD. For example, although the bulk of people within UK Biobank have a BMI lying within the overweight range (25–30 kg/m^2^), reflecting the older age distribution of participants, we still found disproportionately more people with a BMI ≥30 kg/m^2^ among those with asthma compared with asthma-free controls. Excess weight surrounding the chest and abdomen is known to place pressure on the lungs and restrict breathing,^31^ increasing the likelihood of having asthma or developing complications that require hospitalisation. Given the complex interactions between asthma and a variety of characteristics and other health conditions, comprehensive assessments of the overall health profile of asthma patients as part of their routine care would be justified to identify and manage risk factors in those most vulnerable of progressing to more severe forms of asthma and comorbid disease.^32^

It is important to consider our findings in the context of asthma’s wider impact on the UK’s healthcare resources. A recent evaluation of asthma’s financial burden on the NHS assessed the costs associated with the provision of both primary care (prescriptions and consultations) and secondary care (hospital admissions and Accident and Emergency (A&E)) services^5^ and reported healthcare costs being dominated by asthma medication costs at 70%, followed by other primary care (16%) and hospital care (14%) costs. Although most of the economic burden of asthma originates from primary care, we have shown that hospital costs represent a substantive contribution to this burden. A more complete picture of asthma’s wider healthcare costs could be achieved by integrating our estimates of asthma-associated hospital admissions costs with primary care costs and further secondary care costs that include A&E and outpatient care services, ideally from similarly large-cohort retrospective analyses of real-world patient data. Our findings, however, underline the importance of considering the severity of asthma and thus those who are more likely to experience morbidity and greater resource utilisation. There is clear potential for significant savings from preventive interventions that slow progression into more severe forms of disease, preventing hospitalisations, minimising the time spent in hospital and reducing overall expenditure. A more targeted approach to asthma management should reduce these risks and ultimately improve individual patient outcomes.

The main strengths of this study are its use of real-world longitudinal data, the large size of the asthma cohort (25 031) and the ability to identify matched controls from the large UK Biobank cohort. The availability of comprehensive linked hospital admissions data allowed for the assessment of hospital care use and costs, while detailed prescription medication data allowed the identification of separate asthma severity categories and subsequent comparisons of hospital resource use and costs by asthma severity. An accompanying Excel calculator (see online supplemental material 2) illustrates the use of the minimally adjusted models to enable the calculation of predicted mean annual hospital costs according to asthma severity and individual characteristics.

However, some limitations warrant consideration. The definition of active asthma and categorisation of asthma severity relied on the dispense of asthma prescription medications, which does not necessarily correlate with consumption or adherence to said medications.^33^ Some participants may have, therefore, been misclassified into the wrong asthma severity category. Furthermore, we risk oversimplifying asthma severity and underestimating its complexity by classifying patients into only two severity categories. However, prescription medications were self-reported at recruitment into UK Biobank, and lacked information regarding the dosage and quantity of inhaled corticosteroid medications. Thus, we were unable to stratify participants into individual BTS steps and instead used the two broader asthma severity categories. Future studies would benefit from employing more comprehensive categorisation systems that distinguish more nuanced asthma severity categories, in addition to other measures of asthma severity that go beyond medication intensity, such as exacerbations and asthma control status. More generally, there is evidence of a healthy volunteer selection bias in UK Biobank. Recruitment of participants into UK Biobank relied on volunteers: 500 000 participants responded to postal invitations sent to a total of 9.2 million people, making the response rate 5.47%.^34^ It is a well-established phenomenon that those who volunteer for research studies on average tend to be more health conscious and subsequently experience lower rates of most health conditions than non-participants.^35^ A study examining the UK Biobank cohort found that participants were not representative of the general population regarding a number of lifestyle, sociodemographic, physical and health-related characteristics.^36^ They were less likely to be current smokers, to have obesity, to be socioeconomically deprived, and report a lower prevalence of medical conditions, including respiratory disease.^36^ Since our study population is likely healthier than the wider UK population, the overall rates and costs of hospital admissions we report may have been underestimated, limiting generalisability. However, our internal comparisons between asthma and matched controls should provide accurate depictions of the relative differences in hospital care burden between these groups. Finally, the UK Biobank cohort contains a relatively low proportion of patients from ethnic minorities, compromising the generalisability of comparisons made among non-white ethnicities.

## Conclusion

The present study provides a real-world evaluation of the excess hospital care use and costs of adults with asthma in the UK across categories of asthma severity. Asthma clearly places a very large burden on secondary care, with severity significantly affecting the size of this burden. Understanding the scale of hospitalisation use and costs is central to justifying the development and use of novel therapeutic strategies that alleviate the burden of asthma.

## Data Availability

Data may be obtained from a third party and are not publicly available. Data underlining this work may be obtained from the UK Biobank https://www.ukbiobank.ac.uk/ and are not publicly available. Researchers can apply to use the UK Biobank resource. The code (R scripts) used in the analysis for this study are available at https://github.com/anyajacobs/UKBiobank.
