# Supplementary material for: Strong and graded associations between level of asthma severity and all-cause hospital care use and costs in the UK
Source: BMJ Open Respir Res. 2023 Dec 14;10(1):e002003. doi: 10.1136/bmjresp-2023-002003 (PMC10729223; doi:10.1136/bmjresp-2023-002003)
Supplement: Supplementary data [file bmjresp-2023-002003supp001.pdf]

Online Supplemental Material 1

Strong and graded associations between level of asthma severity and all-cause hospital care use and costs in the UK

Supplemental methods

Identifying participants with asthma in UK Biobank

Participants with asthma were required to have doctor-diagnosed asthma and be prescribed asthma medication as per the British Thoracic Society (BTS) steps 1-5[1] at recruitment into UK Biobank. Participants with doctor-diagnosed asthma were identified using the pre-defined asthma algorithm adopted by the UK Biobank.[2] The date of asthma diagnosis had to precede their recruitment date into UK Biobank to be considered an eligible asthma patient (i.e., those who had their first asthma diagnosis after recruitment, during their follow-up period, were excluded). Participants identified with doctor-diagnosed asthma but without report of any asthma medication were excluded as it was not possible to determine whether they had active asthma.

Stratifying asthma patients by asthma severity

Participants were categorised by disease severity into "mild" and "moderate-severe" asthma cohorts according to the type and dosage of asthma medications they were taking at recruitment into UK Biobank. Moderate-to-severe asthma was defined by BTS stage 3-5 criteria, as specified in Shrine et al.[3] Stage 3 involves use of a long-acting β_2 agonist along with an inhaled corticosteroid, stage 4 involves taking a higher dose of inhaled corticosteroid than stage 3 patients in addition to a fourth drug (e.g., leukotriene receptor antagonist, theophylline), while stage 5 involves taking frequent oral corticosteroids, omalizumab, or a combination. Participants taking medication from both mild and moderate-to-severe categories were categorised into the moderate-to-severe population.

Matching algorithm

Participants free from asthma at entry into UK Biobank were randomly selected to act as controls to participants with asthma. For each individual with asthma, 5 asthma-free controls matched for age, sex, ethnicity, and geographic location (defined using the 'assessment centre' variable within UK Biobank) were randomly selected without replacement. Age was a numeric variable, while sex, ethnicity and location were specified as categorical variables.

Matching was conducted separately for mild and moderate-severe asthma patients, producing two asthma-free control cohorts. To identify controls, 5:1 nearest neighbour matching was performed with the Mahalanobis distance and without replacement using R's "MatchIt" package.[4] The quality of matching was confirmed by assessing the balance of matched covariates by computing the standardised mean differences using R's "cobalt" package.[5]

Identifying hospital admissions with a respiratory condition as the primary diagnosis

Hospital admissions were categorised according to the ICD-10 chapter assigned to the primary diagnosis in the first episode of each admission. Hospital admissions were categorised as respiratory if any code from ICD-10 Chapter X: "Diseases of the Respiratory System" [6] was present as the primary diagnosis (i.e., codes J00 to J99).

Identifying hospital admissions with asthma as the primary diagnosis

The definition for an asthma admission was any hospital admission with a primary diagnosis of asthma (ICD-10 codes J45 (Asthma) or J46 (Status asthmaticus)).

Model selection

The comparison of different model specifications revealed that the negative binomial model outperformed zero-inflated negative binomial regression models for hospital admissions and days spent in hospital, and outperformed zero-inflated negative binomial and generalised linear models

Jacobs A, *et al. BMJ Open Respir Res* 2023; 10:e002003. doi: 10.1136/bmjresp-2023-002003

with gaussian distribution and identity link regressions when modelling hospital costs. Negative binomial models produced best model fit in terms of demonstrating largest maximum likelihood statistic. Clustered standard errors were estimated using R's "lmtest" and "sandwich" packages.[7,8]

Costing hospital admissions
Linked Hospital Episode Statistics (HES) data is available for all hospital admissions of UK Biobank participants during study follow-up. These hospital admissions data are organised into hospital episodes. A hospital admission could include one or more hospital episodes. Prior to this study, all hospital episodes were mapped into core Healthcare Resource Groups (HRGs)[9], including core and any unbundled HRGs, using the HRG4+ reference costs grouper.[10] The cost for each hospital admission equalled the sum of the costs of all overlapping episodes. 2018/2019 national schedule of reference costs provided the costs of core HRGs, which vary depending on the class of the episode: "Elective," "Non-elective long stay," "Non-elective short stay," "Day case," or "Regular day or night", and excess bed day costs per HRG, which together were used to cost all hospital episodes.[11]

Jacobs A, *et al. BMJ Open Respir Res* 2023; 10:e002003. doi: 10.1136/bmjresp-2023-002003

Supplemental Table S1. Annual hospital admissions, days spent in hospital, and hospital costs of participants with mild and moderate-severe asthma and of their matched controls, by year of follow-up.

Hospital measure	Mild asthma (n = 19,959) Mean (SD)	Matched asthma-free controls (n = 99,795) Mean (SD)	Moderate-severe asthma (n = 5,072) Mean (SD)	Matched asthma-free controls (n = 25,360) Mean (SD)
Annual hospital admissions				
1	0.36 (2.1)	0.27 (1.6)	0.61 (3.3)	0.27 (0.9)
2	0.40 (2.2)	0.30 (1.7)	0.66 (2.9)	0.31 (1.1)
3	0.43 (2.1)	0.31 (1.7)	0.66 (2.7)	0.33 (1.2)
4	0.45 (2.0)	0.33 (1.7)	0.66 (1.6)	0.35 (1.3)
5	0.47 (2.3)	0.35 (1.9)	0.68 (1.7)	0.36 (1.4)
6	0.49 (2.2)	0.37 (2.0)	0.74 (2.9)	0.40 (1.5)
7	0.51 (1.9)	0.39 (2.0)	0.78 (3.0)	0.43 (1.6)
8	0.54 (1.8)	0.40 (2.0)	0.81 (3.1)	0.45 (2.1)
9	0.55 (2.0)	0.43 (2.0)	0.86 (3.3)	0.47 (1.9)
10	0.58 (2.0)	0.44 (2.1)	0.88 (3.4)	0.49 (2.1)
Annual days in hospital				
1	0.72 (5.2)	0.53 (6.1)	1.49 (7.1)	0.51 (3.9)
2	0.80 (5.1)	0.58 (4.8)	1.71 (8.7)	0.69 (6.4)
3	0.91 (7.1)	0.60 (5.9)	1.56 (6.7)	0.69 (5.0)
4	0.85 (5.2)	0.62 (5.4)	1.98 (10.0)	0.78 (6.9)
5	0.96 (6.2)	0.69 (5.5)	1.78 (11.2)	0.83 (6.2)
6	1.02 (6.1)	0.73 (6.7)	1.97 (9.1)	0.85 (6.2)
7	1.11 (7.9)	0.78 (7.0)	1.98 (9.0)	0.89 (6.0)
8	1.16 (6.7)	0.80 (6.4)	2.32 (11.5)	0.91 (6.1)
9	1.22 (7.5)	0.87 (6.3)	2.22 (9.2)	1.04 (6.6)
10	1.25 (7.2)	0.89 (6.2)	2.22 (9.3)	1.03 (7.4)
Annual hospital costs				
1	£583 (£2,424)	£436 (£2,092)	£1,020 (£3,350)	£447 (£1,835)
2	£657 (£3,219)	£487 (£2,210)	£1,104 (£3,214)	£559 (£2,336)
3	£714 (£4,041)	£511 (£2,384)	£1,086 (£3,231)	£587 (£2,339)
4	£732 (£2,823)	£537 (£2,443)	£1,217 (£3,673)	£625 (£2,581)
5	£776 (£3,131)	£591 (£2,629)	£1,241 (£3,548)	£689 (£2,974)
6	£832 (£2,981)	£630 (£3,104)	£1,401 (£4,515)	£709 (£2,962)
7	£892 (£3,209)	£655 (£2,774)	£1,348 (£3,809)	£758 (£3,075)
8	£932 (£3,332)	£674 (£2,897)	£1,530 (£4,570)	£773 (£3,028)
9	£990 (£3,634)	£747 (£3,273)	£1,619 (£4,756)	£847 (£3,208)
10	£1,040 (£3,785)	£790 (£3,556)	£1,661 (£4,746)	£877 (£3,337)

SD = standard deviation. Controls were matched to participants with mild or moderate-severe asthma based on age, sex, ethnicity and location.

Supplemental Table S2. Annual hospital admissions, days spent in hospital and hospital costs of participants with mild and moderate-severe asthma and of their respective matched control populations. Annual data was calculated from the mean of the first 10 years of follow-up.

**Outcome**	**Mild asthma n = 19,959**		**Matched controls n = 99,795**		**Test for difference**	**Moderate-severe asthma n = 5,072**		**Matched controls n = 25,360**		**Test for difference**
	**Mean**	**(SD)**	**Mean**	**(SD)**		**Mean**	**(SD)**	**Mean**	**(SD)**	
Annual hospital admissions	0.47	(2.06)	0.36	(1.88)	$p < 0.001$	0.72	(2.83)	0.38	(1.54)	$p < 0.001$
Annual days in hospital	0.99	(6.46)	0.70	(6.03)	$p < 0.001$	1.90	(9.27)	0.81	(6.11)	$p < 0.001$
Annual hospital admission costs	£805	(£3,272)	£589	(£2,741)	$p < 0.001$	£1,305	(£3,946)	£679	(£2,782)	$p < 0.001$

SD = standard deviation. Controls were matched to patients with mild or moderate-severe asthma based on age, sex, ethnicity and location. T-tests were used to test for significance between the respective asthma and asthma-free cohort

Jacobs A, *et al. BMJ Open Respir Res* 2023; 10:e002003. doi: 10.1136/bmjresp-2023-002003

Supplemental Table S3. Number of hospital admissions by primary diagnosis chapter (ICD-10) of participants with mild and moderate-severe asthma and of their respective matched control populations. Admissions data are extracted from the 10-year observation period.

ICD-10 chapter	Description	Mild asthma (n = 19,959) N (%)	Matched controls (n = 99,795) N (%)	Moderate-severe asthma (n = 5,072) N (%)	Matched controls (n = 25,360) N (%)
**I**	Certain infectious and parasitic diseases	583 (1.3)	2071 (1.2)	204 (1.3)	574 (1.2)
**II**	Neoplasms	4270 (9.5)	20198 (11.6)	1292 (8.5)	5881 (12.1)
**III**	Diseases of the blood and certain immune disorders	668 (1.5)	2312 (1.3)	237 (1.6)	642 (1. 3)
**IV**	Endocrine, nutritional and metabolic diseases	513 (1.1)	1830 (1.1)	188 (1.2)	489 (1.0)
**V**	Mental and behavioural disorders	185 (0.4)	773 (0.4)	52 (0.3)	205 (0.4)
**VI**	Diseases of the nervous system	1208 (2.7)	4360 (2.5)	320 (2.1)	1152 (2.4)
**VII**	Diseases of the eye and adnexa	2607 (5.8)	11068 (6.4)	948 (6.2)	3356 (6.9)
**VIII**	Diseases of the ear and mastoid process	255 (0.6)	896 (0.5)	87 (0.6)	254 (0.5)
**IX**	Diseases of the circulatory system	2698 (6.0)	11537 (6.6)	1035 (6.8)	3566 (7.3)
**X**	Diseases of the respiratory system	2821 (6.3)	4558 (2.6)	1905 (12.5)	1333 (2.7)
**XI**	Diseases of the digestive system	8741 (19.4)	34387 (19.8)	2694 (17.7)	9437 (19.4)
**XII**	Diseases of the skin and subcutaneous tissue	1036 (2.3)	4528 (2.6)	334 (2.2)	1255 (2.6)
**XIII**	Diseases of the musculoskeletal system and connective tissue	7162 (15.9)	26729 (15.4)	2303 (15.1)	7503 (15.4)
**XIV**	Diseases of the genitourinary system	2749 (6.1)	11778 (6.8)	759 (5.0)	3049 (6.3)
**XV**	Pregnancy, childbirth and the puerperium	30 (0.1)	212 (0.1)	4 (0.0)	26 (0.1)

Jacobs A, *et al. BMJ Open Respir Res* 2023; 10:e002003. doi: 10.1136/bmjresp-2023-002003

**XVI**	Certain conditions originating in the perinatal period	N/A	N/A	N/A	N/A
**XVII**	Congenital malformations and chromosomal abnormalities	87 (0. 2)	315 (0.2)	20 (0.1)	76 (0.2)
**XVIII**	Non-classified symptoms and abnormal clinical findings	5226 (11.6)	19418 (11.2)	1653 (10.8)	5219 (10.7)
**XIX**	Injury, poisoning, consequences of external causes	2066 (4.6)	8091 (4.6)	689 (4.5)	2161 (4.4)
**XX**	External causes of morbidity	N/A	N/A	N/A	N/A
**XXI**	Factors influencing health status and contact with health services	1986 (4.4)	8764 (5.0)	513 (3.4)	2463 (5.1)
**XXII**	Codes for special purposes	N/A	5 (0.0)	N/A	N/A
	**TOTAL (N)**	188,619	944,853	46,980	239,582

REGRESSION OUTPUTS: mild asthma vs no asthma

Supplemental Table S4. Negative binomial regression models of hospital admissions, days spent in hospital and hospital costs for people with mild asthma compared to controls matched on age, sex, ethnicity and location. All models were further adjusted for age, sex, ethnicity and location (minimally adjusted).

	**Hospital admissions IRR (95% CI)**	**Days in hospital IRR (95% CI)**	**Hospital costs IRR (95% CI)**
**Intercept**	0.25*** (0.24, 0.26)	0.47*** (0.42, 0.52)	416.69*** (381.93, 432.32)
**Mild asthma (Ref: No asthma)**	1.36*** (1.28, 1.40)	1.43*** (1.35, 1.51)	1.36*** (1.31, 1.41)
**Follow-up year (Ref: 1)**			
2	1.10*** (1.07, 1.13)	1.04 (0.95, 1.15)	1.09*** (1.06, 1.13)
3	1.14*** (1.12, 1.19)	1.09 (0.99, 1.20)	1.15*** (1.12, 1.20)
4	1.22*** (1.18, 1.26)	1.09 (1.00, 1.20)	1.20*** (1.17, 1.25)
5	1.30*** (1.25, 1.35)	1.19** (1.09, 1.30)	1.31*** (1.26, 1.36)
6	1.38*** (1.32, 1.43)	1.31*** (1.18, 1.45)	1.40*** (1.35, 1.46)
7	1.44*** (1.37, 1.49)	1.36*** (1.24, 1.49)	1.45*** (1.40, 1.51)
8	1.49*** (1.43, 1.55)	1.41*** (1.28, 1.54)	1.50*** (1.45, 1.56)
9	1.59*** (1.51, 1.65)	1.53*** (1.39, 1.68)	1.64*** (1.58, 1.70)
10	1.64*** (1.57, 1.71)	1.58*** (1.44, 1.74)	1.74*** (1.67, 1.81)
**Age at recruitment (centred at 56), per year**	1.04*** (1.04, 1.04)	1.05*** (1.05, 1.06)	1.05*** (1.04, 1.05)
**Male sex (Ref: female)**	1.02 (0.98, 1.06)	1.16*** (1.11, 1.21)	1.02 (0.99, 1.05)
**Ethnicity (Ref: White)**			
Black	1.30** (1.07, 1.58)	1.61*** (1.33, 1.93)	1.22*** (1.09, 1.36)
South Asian	1.18** (1.05, 1.34)	1.27* (1.05, 1.55)	1.11* (1.00, 1.22)
Other	1.11 (0.99, 1.26)	1.08 (0.95, 1.24)	1.04 (0.95, 1.14)
**Log Likelihood**	-821529.98	-890496.32	-2694315.17

Incident Rate Ratios (IRRs) are shown for each variable, derived from exponentiating regression coefficients. Confidence intervals are in brackets. Age at recruitment was centred at 56-years. Reference ethnicity was white. All models were further adjusted for location (not displayed). Significance: *** p<0.001; ** p <0.01; * p<0.05.

Supplemental Table S5. Negative binomial regression models of hospital admissions, days spent in hospital and hospital costs for people with mild asthma compared to controls matched on age, sex, ethnicity and location. All models were further adjusted for age, sex, ethnicity, location (factors already matched for), and socioeconomic deprivation (intermediately adjusted).

	**Hospital admissions IRR (95% CI)**	**Days in hospital IRR (95% CI)**	**Hospital costs IRR (95% CI)**
**Intercept**	0.23*** (0.21, 0.24)	0.36*** (0.30, 0.41)	371.65*** (350.95, 393.58)
**Mild asthma (Ref: No asthma)**	1.32*** (1.26, 1.37)	1.36*** (1.29, 1.43)	1.32*** (1.27, 1.38)
**Follow-up year (Ref: 1)**			
2	1.10*** (1.08, 1.13)	1.06 (0.98, 1.15)	1.10*** (1.06, 1.14)
3	1.15*** (1.12, 1.19)	1.09 (1.00, 1.18)	1.16*** (1.12, 1.20)
4	1.23*** (1.18, 1.27)	1.10 (1.01, 1.19)	1.21*** (1.17, 1.26)
5	1.30*** (1.26, 1.35)	1.20*** (1.11, 1.30)	1.31*** (1.26, 1.36)
6	1.38*** (1.33, 1.44)	1.31*** (1.20, 1.44)	1.41*** (1.35, 1.47)
7	1.43*** (1.38, 1.49)	1.37*** (1.26, 1.50)	1.46*** (1.41, 1.51)
8	1.50*** (1.44, 1.56)	1.42*** (1.31, 1.55)	1.51*** (1.46, 1.57)
9	1.59*** (1.52, 1.66)	1.56*** (1.42, 1.70)	1.64*** (1.58, 1.71)
10	1.64*** (1.58, 1.72)	1.61*** (1.47, 1.76)	1.75*** (1.68, 1.82)
**Age at recruitment (centred at 56), per year**	1.04*** (1.04, 1.04)	1.06*** (1.05, 1.06)	1.05*** (1.05, 1.05)
**Male sex (Ref: female)**	1.02 (0.98, 1.05)	1.13*** (1.09, 1.19)	1.01 (0.98, 1.04)
**Ethnicity (Ref: White)**			
Black	1.17 (0.97, 1.41)	1.31** (1.09, 1.58)	1.09 (0.98, 1.21)
South Asian	1.14* (1.00, 1.3)	1.21 (0.99, 1.47)	1.07 (0.97, 1.18)
Other	1.05 (0.94, 1.18)	0.97 (0.85, 1.11)	0.97 (0.89, 1.07)
**Deprivation (Townsend) (Ref: Quintile 1, least deprived)**			
Quintile 2	1.09** (1.03, 1.14)	1.18*** (1.11, 1.26)	1.07*** (1.03, 1.11)
Quintile 3	1.12*** (1.06, 1.19)	1.33*** (1.24, 1.42)	1.14*** (1.09, 1.19)
Quintile 4	1.23*** (1.18, 1.29)	1.61*** (1.51, 1.72)	1.31*** (1.25, 1.37)
Quintile 5	1.47*** (1.38, 1.56)	2.21*** (2.04, 2.40)	1.53*** (1.46, 1.61)
**Log Likelihood**	-820548.07	-887698.72	-2694088.16

Incident Rate Ratios (IRRs) are shown for each variable, derived from exponentiating regression coefficients. Confidence intervals are in brackets. Age at recruitment was centred at 56-years. Reference ethnicity was white and reference deprivation was Townsend quintile 1 (least deprived). All models were further adjusted for location (not displayed).
Significance: *** p<0.001; ** p<0.01; * p<0.05.

Supplemental Table S6. Negative binomial regression models of hospital admissions, days spent in hospital and hospital costs for people with mild asthma compared to controls matched on age, sex, ethnicity and location. All models were further adjusted for age, sex, ethnicity, location, socioeconomic deprivation, smoking status, BMI category and comorbidities (fully adjusted).

	**Hospital admissions IRR (95% CI)**	**Days in hospital IRR (95% CI)**	**Hospital costs IRR (95% CI)**
**Intercept**	0.16*** (0.16, 0.17)	0.23*** (0.21, 0.25)	224.01*** (240.29, 268.51)
**Mild asthma (Ref: No asthma)**	1.24*** (1.20, 1.28)	1.21** (1.15, 1.27)	1.21*** (1.17, 1.25)
**Follow-up year (Ref: 1)**			
2	1.12*** (1.09, 1.15)	1.09* (1.01, 1.18)	1.12*** (1.08, 1.16)
3	1.18*** (1.15, 1.22)	1.14* (1.05, 1.24)	1.19*** (1.14, 1.23)
4	1.27*** (1.23, 1.31)	1.17*** (1.08, 1.27)	1.25*** (1.20, 1.29)
5	1.36*** (1.31, 1.40)	1.27*** (1.18, 1.37)	1.35*** (1.30, 1.40)
6	1.44*** (1.40, 1.49)	1.42*** (1.29, 1.55)	1.47*** (1.41, 1.53)
7	1.50*** (1.45, 1.55)	1.47*** (1.35, 1.59)	1.52*** (1.46, 1.57)
8	1.58*** (1.53, 1.63)	1.52*** (1.41, 1.65)	1.57*** (1.52, 1.63)
9	1.69*** (1.63, 1.74)	1.70*** (1.57, 1.86)	1.71*** (1.65, 1.78)
10	1.75*** (1.69, 1.81)	1.74*** (1.61, 1.89)	1.81*** (1.74, 1.88)
**Age at recruitment (centred at 56), per year**	1.03*** (1.03, 1.03)	1.05*** (1.05, 1.05)	1.04*** (1.04, 1.04)
**Male sex (Ref: female)**	0.99 (0.97, 1.02)	1.11*** (1.06, 1.16)	0.98 (0.95, 1.01)
**Ethnicity (Ref: White)**			
Black	1.07 (0.96, 1.21)	1.28* (1.05, 1.56)	1.04 (0.93, 1.15)
South Asian	1.12** (1.03, 1.21)	1.25 (0.98, 1.58)	1.05 (0.95, 1.16)
Other	1.04 (0.95, 1.13)	0.99 (0.87, 1.14)	0.98 (0.9, 1.07)
**Deprivation (Townsend) (Ref: Quintile 1, least deprived)**			
Quintile 2	1.04* (1.00, 1.09)	1.11** (1.04, 1.18)	1.04* (1.00, 1.08)
Quintile 3	1.04 (1.00, 1.08)	1.17*** (1.09, 1.25)	1.06** (1.02, 1.10)
Quintile 4	1.11*** (1.07, 1.16)	1.30*** (1.22, 1.39)	1.16*** (1.10, 1.21)
Quintile 5	1.18*** (1.12, 1.23)	1.50*** (1.4, 1.62)	1.21*** (1.16, 1.27)
**Smoking status (Ref: No smoker)**			
Current smoker	1.27*** (1.22, 1.33)	1.57*** (1.46, 1.70)	1.38*** (1.32, 1.45)
Previous smoker	1.09*** (1.06, 1.12)	1.08** (1.03, 1.13)	1.11*** (1.08, 1.14)
**BMI category (Ref: 18.5-25)**			
<18.5	1.60* (1.11, 2.32)	1.69*** (1.33, 2.14)	1.64** (1.17, 2.30)
25-30	1.07*** (1.04, 1.11)	1.06* (1.01, 1.12)	1.11*** (1.07, 1.15)

Jacobs A, *et al. BMJ Open Respir Res* 2023; 10:e002003. doi: 10.1136/bmjresp-2023-002003

30-35	1.18*** (1.13, 1.22)	1.28*** (1.21, 1.37)	1.27*** (1.23, 1.32)
35-40	1.31*** (1.23, 1.40)	1.53*** (1.37, 1.70)	1.45*** (1.38, 1.53)
40+	1.48*** (1.36, 1.61)	1.98*** (1.76, 2.23)	1.74*** (1.61, 1.88)
**Comorbidity**			
COPD	1.46*** (1.34, 1.59)	1.72*** (1.55, 1.89)	1.58*** (1.43, 1.74)
Diabetes	1.30*** (1.22, 1.39)	1.59*** (1.47, 1.73)	1.39*** (1.31, 1.47)
Hypertension	1.21*** (1.17, 1.25)	1.25*** (1.19, 1.31)	1.23*** (1.19, 1.26)
Myocardial infarction	1.38*** (1.26, 1.50)	1.57*** (1.44, 1.72)	1.53*** (1.41, 1.66)
Stroke	1.41*** (1.23, 1.61)	1.81*** (1.58, 2.07)	1.47*** (1.36, 1.59)
Cancer	1.89*** (1.80, 1.98)	2.02*** (1.89, 2.15)	1.88*** (1.79, 1.97)
Sleep apnoea	1.16 (0.97, 1.39)	1.25 (0.88, 1.78)	1.24* (1.04, 1.48)
CKD	2.58*** (1.91, 3.50)	2.02*** (1.66, 2.45)	1.66*** (1.45, 1.92)
PVD	1.78*** (1.52, 2.08)	1.80*** (1.61, 2.03)	1.65*** (1.50, 1.83)
Mental disorder	1.27*** (1.22, 1.32)	1.83*** (1.67, 2.01)	1.32*** (1.27, 1.38)
**Log Likelihood**	-800211.91	-864725.08	-2659167.77

Incident Rate Ratios (IRRs) are shown for each variable, derived from exponentiating regression coefficients. Confidence intervals are in brackets. Age at recruitment was centred at 56-years. Reference ethnicity was white, reference deprivation was Townsend quintile 1 (least deprived), reference smoking status was 'never smoked' and reference BMI category was 18.5-25. All models were further adjusted for location (not displayed). COPD = Chronic Obstructive Pulmonary Disease. CKD = chronic kidney disease. PVD = peripheral arterial disease. Significance: *** p<0.001; ** p<0.01; * p<0.05.

REGRESSION MODELS: moderate-severe asthma vs no asthma

Supplemental Table S7. Negative binomial regression models of hospital admissions, days spent in hospital and hospital costs for people with moderate-severe asthma compared to controls matched on age, sex, ethnicity and location. All models were further adjusted for age, sex, ethnicity, and location (minimally adjusted).

	**Hospital admissions IRR (95% CI)**	**Days in hospital IRR (95% CI)**	**Hospital costs IRR (95% CI)**
**Intercept**	0.026*** (0.24, 0.28)	0.46*** (0.38, 0.55)	425.10*** (391.67, 461.39)
**Moderate-severe asthma (Ref: No asthma)**	1.93*** (1.81, 2.07)	2.42*** (2.24, 2.62)	1.98*** (1.88, 2.08)
**Follow-up year (Ref: 1)**			
2	1.14*** (1.09, 1.19)	1.28*** (1.14, 1.43)	1.20*** (1.14, 1.27)
3	1.20*** (1.14, 1.26)	1.23*** (1.11, 1.37)	1.25*** (1.18, 1.33)
4	1.26*** (1.19, 1.33)	1.38*** (1.23, 1.56)	1.32*** (1.24, 1.40)
5	1.31*** (1.24, 1.39)	1.55*** (1.39, 1.73)	1.49*** (1.39, 1.59)
6	1.42*** (1.33, 1.51)	1.49*** (1.33, 1.67)	1.50*** (1.41, 1.61)
7	1.52*** (1.44, 1.62)	1.56*** (1.40, 1.73)	1.58*** (1.48, 1.68)
8	1.58*** (1.48, 1.68)	1.63*** (1.46, 1.81)	1.63*** (1.54, 1.74)
9	1.64*** (1.54, 1.75)	1.83*** (1.65, 2.03)	1.75*** (1.65, 1.87)
10	1.72*** (1.61, 1.84)	1.88*** (1.66, 2.14)	1.85*** (1.73, 1.98)
**Age at recruitment (centred at 56), per year**	1.04*** (1.03, 1.04)	1.05*** (1.04, 1.06)	1.04*** (1.04, 1.05)
**Male sex (Ref: female)**	1.10*** (1.05, 1.15)	1.29*** (1.20, 1.40)	1.14*** (1.09, 1.19)
**Ethnicity (Ref: White)**			
Black	1.20 (0.91, 1.57)	1.07 (0.79, 1.46)	1.10 (0.86, 1.41)
South Asian	1.27** (1.10, 1.46)	1.05 (0.83, 1.33)	1.10 (0.93, 1.31)
Other	0.95 (0.83, 1.09)	0.94 (0.73, 1.21)	0.89 (0.76, 1.04)
**Log Likelihood**	-230626.44	-259180.31	-780216.07

Incident Rate Ratios (IRRs) are shown for each variable, derived from exponentiating regression coefficients. Confidence intervals are in brackets. Age at recruitment was centred at 56-years. Reference ethnicity was white. All models were further adjusted for location (not displayed). Significance: *** p<0.001; ** p<0.01; * p<0.05.

Jacobs A, *et al. BMJ Open Respir Res* 2023; 10:e002003. doi: 10.1136/bmjresp-2023-002003

Supplemental Table S8. Negative binomial regression models of hospital admissions, days spent in hospital and hospital costs for people with moderate-severe asthma compared to controls matched on age, sex, ethnicity and location. All models were further adjusted for age, sex, ethnicity, location (factors already matched for), and socioeconomic deprivation (intermediately adjusted).

	**Hospital admissions IRR (95% CI)**	**Days in hospital IRR (95% CI)**	**Hospital costs IRR (95% CI)**
**Intercept**	0.23*** (0.21, 0.25)	0.36*** (0.30, 0.44)	373.65*** (342.91, 407.16)
**Moderate-severe asthma (Ref: No asthma)**	1.84*** (1.73, 1.96)	2.21*** (2.04, 2.40)	1.86*** (1.76, 1.96)
**Follow-up year (Ref: 1)**			
2	1.14*** (1.10, 1.20)	1.28*** (1.14, 1.43)	1.20*** (1.14, 1.27)
3	1.20*** (1.14, 1.26)	1.24*** (1.11, 1.37)	1.25*** (1.18, 1.33)
4	1.26*** (1.20, 1.33)	1.38*** (1.23, 1.55)	1.32*** (1.25, 1.41)
5	1.32*** (1.25, 1.40)	1.58*** (1.41, 1.76)	1.50*** (1.40, 1.60)
6	1.42*** (1.34, 1.51)	1.50*** (1.34, 1.67)	1.50*** (1.41, 1.61)
7	1.53*** (1.45, 1.63)	1.57*** (1.41, 1.75)	1.59*** (1.49, 1.70)
8	1.59*** (1.49, 1.70)	1.65*** (1.48, 1.83)	1.64*** (1.54, 1.74)
9	1.65*** (1.55, 1.76)	1.83*** (1.64, 2.03)	1.76*** (1.65, 1.87)
10	1.72*** (1.62, 1.84)	1.91*** (1.70, 2.16)	1.87*** (1.75, 2.01)
**Age at recruitment (centred at 56), per year**	1.04*** (1.03, 1.04)	1.05*** (1.05, 1.06)	1.04*** (1.04, 1.05)
**Male sex (Ref: female)**	1.09*** (1.04, 1.14)	1.27*** (1.18, 1.37)	1.13*** (1.08, 1.18)
**Ethnicity (Ref: White)**			
Black	1.07 (0.81, 1.41)	0.89 (0.65, 1.23)	0.98 (0.76, 1.25)
South Asian	1.22** (1.06, 1.41)	0.97 (0.77, 1.23)	1.06 (0.89, 1.25)
Other	0.90 (0.79, 1.03)	0.83 (0.66, 1.05)	0.84* (0.72, 0.98)
**Deprivation (Townsend) (Ref: Quintile 1, least deprived)**			
Quintile 2	1.06 (1.00, 1.13)	1.21*** (1.09, 1.35)	1.12*** (1.05, 1.19)
Quintile 3	1.11** (1.03, 1.19)	1.30*** (1.17, 1.44)	1.15*** (1.07, 1.22)
Quintile 4	1.25*** (1.17, 1.34)	1.60*** (1.41, 1.82)	1.35*** (1.26, 1.45)
Quintile 5	1.55*** (1.42, 1.68)	2.20*** (1.96, 2.48)	1.65*** (1.53, 1.77)
**Log Likelihood**	-230216.82	-258386.09	-780125.08

Incident Rate Ratios (IRRs) are shown for each variable, derived from exponentiating regression coefficients. Confidence intervals are in brackets. Age at recruitment was centred at 56-years. Reference ethnicity was white and reference deprivation was Townsend quintile 1 (least deprived). All models were further adjusted for location (not displayed). Significance: *** p<0.001; ** p<0.01; * p<0.05.

Supplemental Table S9. Negative binomial regression models of hospital admissions, days spent in hospital and hospital costs for people with moderate-severe asthma compared to controls matched on age, sex, ethnicity and location. All models were further adjusted for age, sex, ethnicity, location, socioeconomic deprivation, smoking status, BMI category and comorbidities (fully adjusted).

	**Hospital admissions IRR (95% CI)**	**Days in hospital IRR (95% CI)**	**Hospital costs IRR (95% CI)**
**Intercept**	0.17***<br>(0.16, 0.19)	0.24***<br>(0.19, 0.29)	225.28***<br>(232.79, 279.93)
**Moderate-severe asthma (Ref: No asthma)**	1.50*** (1.42, 1.58)	1.65** (1.51, 1.80)	1.50*** (1.43, 1.59)
**Follow-up year (Ref: 1)**			
2	1.16*** (1.11, 1.21)	1.25*** (1.12, 1.39)	1.22*** (1.15, 1.29)
3	1.22*** (1.17, 1.28)	1.27*** (1.14, 1.41)	1.28*** (1.21, 1.37)
4	1.30*** (1.24, 1.37)	1.38*** (1.23, 1.55)	1.36*** (1.27, 1.44)
5	1.37*** (1.30, 1.45)	1.63*** (1.45, 1.84)	1.54*** (1.44, 1.64)
6	1.48*** (1.40, 1.56)	1.57*** (1.40, 1.76)	1.59*** (1.48, 1.71)
7	1.59*** (1.50, 1.67)	1.66*** (1.49, 1.86)	1.67*** (1.56, 1.79)
8	1.66*** (1.56, 1.77)	1.76*** (1.58, 1.96)	1.73*** (1.63, 1.84)
9	1.74*** (1.64, 1.85)	1.96*** (1.76, 2.19)	1.87*** (1.75, 1.99)
10	1.80*** (1.70, 1.91)	2.00*** (1.77, 2.25)	1.96*** (1.83, 2.11)
**Age at recruitment (centred at 56), per year**	1.03*** (1.03, 1.03)	1.05*** (1.04, 1.05)	1.04*** (1.03, 1.04)
**Male sex (Ref: female)**	1.04 (1.00, 1.09)	1.22*** (1.13, 1.31)	1.07** (1.02, 1.12)
**Ethnicity (Ref: White)**			
Black	1.11 (0.84, 1.47)	0.94 (0.68, 1.30)	1.01 (0.77, 1.32)
South Asian	1.28** (1.11, 1.47)	0.97 (0.79, 1.20)	1.12 (0.94, 1.33)
Other	0.93 (0.81, 1.06)	0.82 (0.66, 1.01)	0.86 (0.74, 1.01)
**Deprivation (Townsend) (Ref: Quintile 1, least derived)**			
Quintile 2	1.02 (0.96, 1.08)	1.12* (1.02, 1.24)	1.06* (1.00, 1.13)
Quintile 3	1.04 (0.97, 1.10)	1.18** (1.06, 1.30)	1.06 (0.99, 1.13)
Quintile 4	1.12*** (1.05, 1.20)	1.31*** (1.17, 1.46)	1.17*** (1.09, 1.25)
Quintile 5	1.24*** (1.15, 1.33)	1.57*** (1.39, 1.77)	1.27*** (1.19, 1.37)
**Smoking status**			
Current smoker	1.33*** (1.24, 1.43)	1.59*** (1.41, 1.80)	1.45*** (1.34, 1.56)
Previous smoker	1.14*** (1.09, 1.20)	1.11** (1.03, 1.19)	1.15*** (1.10, 1.20)
**BMI category (Ref: 18.5-25)**			
<18.5	1.57 (0.95, 2.60)	1.82* (1.04, 3.18)	1.21 (0.95, 1.54)
25-30	1.08** (1.02, 1.14)	1.03 (0.95, 1.13)	1.11*** (1.05, 1.17)
30-35	1.13*** (1.06, 1.20)	1.06 (0.97, 1.17)	1.21*** (1.13, 1.28)

Jacobs A, *et al. BMJ Open Respir Res* 2023; 10:e002003. doi: 10.1136/bmjresp-2023-002003

35-40	1.27*** (1.15, 1.39)	1.61*** (1.32, 1.98)	1.46*** (1.32, 1.62)
40+	1.38*** (1.20, 1.58)	1.84*** (1.53, 2.22)	1.70*** (1.50, 1.92)
**Comorbidity**			
COPD	1.57*** (1.41, 1.75)	1.87*** (1.64, 2.12)	1.63*** (1.48, 1.80)
Diabetes	1.24*** (1.15, 1.33)	1.57*** (1.39, 1.78)	1.36*** (1.26, 1.47)
Hypertension	1.18*** (1.13, 1.24)	1.33*** (1.23, 1.44)	1.24*** (1.19, 1.30)
Myocardial infarction	1.29*** (1.17, 1.42)	1.37*** (1.21, 1.55)	1.32*** (1.20, 1.44)
Stroke	1.35*** (1.19, 1.53)	1.99*** (1.64, 2.42)	1.42*** (1.25, 1.61)
Cancer	1.79*** (1.66, 1.92)	1.94*** (1.73, 2.18)	1.78*** (1.66, 1.92)
Sleep apnoea	1.02 (0.86, 1.21)	0.96 (0.70, 1.32)	1.01 (0.82, 1.24)
CKD	1.50** (1.12, 2.02)	1.49*** (1.18, 1.88)	1.30** (1.11, 1.53)
PVD	1.50*** (1.34, 1.67)	1.65*** (1.37, 1.99)	1.56*** (1.38, 1.76)
Mental disorder	1.26*** (1.18, 1.35)	1.75*** (1.52, 2.03)	1.30*** (1.21, 1.40)
**Log Likelihood**	-224911.95	-251596.33	-769545.59

Incident Rate Ratios (IRRs) are shown for each variable, derived from exponentiating regression coefficients. Confidence intervals are in brackets. Age at recruitment was centred at 56-years. Reference ethnicity was white, reference deprivation was Townsend quintile 1 (least deprived), reference smoking status was 'never smoked' and reference BMI category was 18.5-25. All models were further adjusted for location (not displayed). COPD = Chronic Obstructive Pulmonary Disease. CKD = chronic kidney disease. PVD = peripheral arterial disease. Significance: *** p<0.001; ** p<0.01; * p<0.05.

Jacobs A, *et al. BMJ Open Respir Res* 2023; 10:e002003. doi: 10.1136/bmjresp-2023-002003

Supplemental Table S10. Impact of asthma on hospital outcomes across years of follow-up. Negative binomial regression models of hospital admissions, days spent in hospital and hospital costs for people with asthma (mild and moderate-severe) compared to controls matched on age, sex, ethnicity and location, when follow-up year is a count variable. Incident Rate Ratios are shown, derived from exponentiating regression coefficients. Models with different levels of adjustment are compared.

	**MILD ASTHMA IRR (95% CI)**			**MODERATE-SEVERE ASTHMA IRR (95% CI)**		
**HOSPITAL ADMISSIONS**	Minimally adjusted	Intermediately adjusted	Fully adjusted	Minimally adjusted	Intermediately adjusted	Fully adjusted
**Intercept**	0.25*** (0.23, 0.26)	0.22*** (0.21, 0.23)	0.16*** (0.15, 0.17)	0.25*** (0.23, 0.27)	0.23*** (0.21, 0.25)	0.17*** (0.15, 0.19)
**Asthma**	1.38*** (1.27, 1.50)	1.35*** (1.24, 1.46)	1.24*** (1.17, 1.31)	2.21*** (1.87, 2.40)	2.00*** (1.77, 2.26)	1.60*** (1.46, 1.76)
**Follow-up year**	1.06*** (1.05, 1.06)	1.06*** (1.05, 1.06)	1.06*** (1.06, 1.07)	1.06*** (1.06, 1.07)	1.06*** (1.06, 1.07)	1.07*** (1.06, 1.07)
**Asthma * follow-up year**	1.00 (0.98, 1.01)	1.00 (0.99, 1.01)	1.00 (0.99, 1.01)	0.98 (0.96, 1.00)	0.99 (0.96, 1.00)	0.99 (0.97, 1.00)
**HOSPITAL DAYS**	Minimally adjusted	Intermediately adjusted	Fully adjusted	Minimally adjusted	Intermediately adjusted	Fully adjusted
**Intercept**	0.43*** (0.40, 0.47)	0.34*** (0.31, 0.37)	0.22*** (0.20, 0.24)	0.47*** (0.39, 0.57)	0.38*** (0.32, 0.46)	0.24*** (0.20, 0.30)
**Asthma**	1.40*** (1.25, 1.56)	1.34*** (1.21, 1.48)	1.23*** (1.11, 1.36)	2.69*** (2.34, 3.09)	2.37*** (2.06, 2.73)	1.79*** (1.55, 2.07)
**Follow-up year**	1.05*** (1.05, 1.06)	1.06*** (1.05, 1.06)	1.07*** (1.06, 1.07)	1.07*** (1.05, 1.08)	1.07*** (1.05, 1.08)	1.08*** (1.06, 1.09)
**Asthma * follow-up year**	1.00 (0.99, 1.02)	1.00 (0.99, 1.02)	1.00 (0.98, 1.01)	0.98 (0.96, 1.00)	0.99 (0.96, 1.01)	0.98 (0.96, 1.00)
**HOSPITAL COSTS**	Minimally adjusted	Intermediately adjusted	Fully adjusted	Minimally adjusted	Intermediately adjusted	Fully adjusted
**Intercept**	400.70*** (380, 422)	375.33*** (338, 378)	246.73*** (234, 260)	432.39*** (399, 467)	380.89*** (350, 415)	259.66*** (237, 284)
**Asthma**	1.36*** (1.27, 1.46)	1.33*** (1.24, 1.44)	1.21*** (1.13, 1.28)	2.08*** (1.90, 2.27)	1.93*** (1.77, 2.11)	1.58*** (1.44, 1.74)
**Follow-up year**	1.06*** (1.06, 1.07)	1.06*** (1.06, 1.07)	1.07*** (1.06, 1.07)	1.07*** (1.06, 1.07)	1.07*** (1.06, 1.07)	1.07*** (1.07, 1.08)
**Asthma * follow-up year**	1.00 (0.99, 1.01)	1.00 (0.99, 1.01)	1.00 (0.99, 1.01)	0.99 (0.98, 1.00)	0.99 (0.98, 1.01)	0.99 (0.98, 1.00)

Incident Rate Ratios (IRRs) are shown for each variable, derived from exponentiating regression coefficients. Confidence intervals are in brackets. Age at recruitment was centred at 56-years. Levels of model adjustment: minimally adjusted models were adjusted for the matching covariates (age, sex, ethnicity & location); intermediately adjusted models were adjusted for socioeconomic deprivation in addition to the matching covariates; fully adjusted models were adjusted for deprivation, smoking status, BMI category and comorbidities in addition to the matching covariates. Significance: *** p<0.001; ** p<0.01; * p<0.05.

Jacobs A, *et al. BMJ Open Respir Res* 2023; 10:e002003. doi: 10.1136/bmjresp-2023-002003

Supplemental Table S11. Impact of asthma on hospital outcomes across quintiles of socioeconomic deprivation. Negative binomial regression models of hospital admissions, days spent in hospital and hospital costs for people with asthma (mild and moderate-severe) compared to controls matched on age, sex, ethnicity and location. An interaction term between deprivation (as the quintile number) and asthma is included in all regressions. Incident Rate Ratios are shown, derived from exponentiating regression coefficients. Models with different levels of adjustment are compared.

	**MILD ASTHMA IRR (95% CI)**			**MODERATE-SEVERE ASTHMA IRR (95% CI)**		
**HOSPITAL ADMISSIONS**	Minimally adjusted	Intermediately adjusted	Fully adjusted	Minimally adjusted	Intermediately adjusted	Fully adjusted
**Intercept**	0.28*** (0.27, 0.30)	0.28*** (0.27, 0.30)	0.22*** (0.21, 0.24)	0.30*** (0.27, 0.33)	0.30*** (0.28, 0.33)	0.24*** (0.21, 0.26)
**Asthma**	1.13** (1.04, 1.23)	1.13** (1.04, 1.23)	1.14*** (1.06, 1.21)	1.51*** (1.34, 1.7)	1.51*** (1.34, 1.7)	1.40*** (1.26, 1.56)
**Townsend quintile**	1.07*** (1.06, 1.09)	1.07*** (1.06, 1.09)	1.03*** (1.02, 1.05)	1.08*** (1.06, 1.1)	1.08*** (1.06, 1.1)	1.05*** (1.03, 1.07)
**Asthma * Townsend quintile**	1.06*** (1.03, 1.09)	1.06*** (1.03, 1.09)	1.03** (1.01, 1.06)	1.07** (1.03, 1.12)	1.07** (1.03, 1.12)	1.02 (0.99, 1.06)
**HOSPITAL DAYS**	Minimally adjusted	Intermediately adjusted	Fully adjusted	Minimally adjusted	Intermediately adjusted	Fully adjusted
**Intercept**	0.39*** (0.36, 0.42)	0.39*** (0.36, 0.42)	0.29*** (0.27, 0.31)	0.46*** (0.39, 0.54)	0.45*** (0.38, 0.53)	0.33*** (0.27, 0.39)
**Asthma**	1.19** (1.07, 1.33)	1.19** (1.07, 1.33)	1.15** (1.04, 1.26)	1.95*** (1.64, 2.32)	1.95*** (1.64, 2.32)	1.62*** (1.38, 1.91)
**Townsend quintile**	1.18*** (1.16, 1.21)	1.18*** (1.16, 1.21)	1.10*** (1.08, 1.12)	1.18*** (1.14, 1.22)	1.18*** (1.14, 1.22)	1.12*** (1.08, 1.16)
**Asthma * Townsend quintile**	1.05** (1.01, 1.09)	1.05** (1.01, 1.09)	1.02 (1.09, 1.06)	1.05 (0.99, 1.1)	1.05 (0.99, 1.1)	1.00 (0.95, 1.06)
**HOSPITAL COSTS**	Minimally adjusted	Intermediately adjusted	Fully adjusted	Minimally adjusted	Intermediately adjusted	Fully adjusted
**Intercept**	475.44*** (433, 488)	460.08*** (432, 486)	340.91*** (323, 360)	498.42*** (459, 542)	495.73*** (456, 539)	370.68*** (339, 406)
**Asthma**	1.20*** (1.10, 1.30)	1.20*** (1.10, 1.30)	1.17*** (1.10, 1.25)	1.59*** (1.42, 1.77)	1.59*** (1.42, 1.77)	1.42*** (1.28, 1.59)
**Townsend quintile**	1.09*** (1.08, 1.11)	1.09*** (1.08, 1.11)	1.05*** (1.03, 1.06)	1.10*** (1.08, 1.12)	1.10*** (1.08, 1.12)	1.06*** (1.04, 1.08)
**Asthma * Townsend quintile**	1.04** (1.01, 1.07)	1.04** (1.01, 1.07)	1.01 (1.99, 1.04)	1.06** (1.02, 1.0)	1.06** (1.02, 1.0)	1.02 (0.98, 1.05)

Incident Rate Ratios (IRRs) are shown for each variable, derived from exponentiating regression coefficients. Confidence intervals are in brackets. Levels of model adjustment: minimally adjusted models were adjusted for the matching covariates (age, sex, ethnicity & location); intermediately adjusted models were adjusted for socioeconomic deprivation in addition to the matching covariates; fully adjusted models were adjusted for deprivation, smoking status, BMI category and comorbidities in addition to the matching covariates. Significance: *** p<0.001; ** p<0.01; * p<0.05.

Supplemental Table S12. Impact of asthma on the rates of hospital admissions by primary diagnosis of admission. Negative binomial regression models of hospital admissions, stratified by ICD-10 chapter of primary diagnosis of admission, for people with mild and moderate-severe asthma compared to controls. Models with different levels of adjustment are compared.

ICD-10 CHAPTER	DESCRIPTION	MILD ASTHMA IRR (95% CI)			MODERATE-SEVERE ASTHMA IRR (95% CI)		
		Minimally adjusted	Intermediately adjusted	Fully adjusted	Minimally adjusted	Intermediately adjusted	Fully adjusted
**I**	Certain infectious and parasitic diseases	1.55*** (1.40, 1.71)	1.51*** (1.37, 1.67)	1.42*** (1.28, 1.58)	2.31*** (2.01, 2.66)	2.22*** (1.92, 2.56)	1.86*** (1.59, 2.19)
**II**	Neoplasms	1.09 (0.99, 1.20)	1.08 (0.98, 1.20)	1.09 (0.98, 1.21)	1.23** (1.06, 1.43)	1.21** (1.04, 1.41)	1.09 (0.92, 1.29)
**III**	Diseases of the blood and certain immune disorders	2.03*** (1.41, 2.92)	2.04*** (1.39, 2.99)	1.56** (1.14, 2.14)	2.25*** (1.61, 3.14)	2.15*** (1.52, 3.02)	1.52* (1.07, 2.16)
**IV**	Endocrine, nutritional and metabolic diseases	1.38** (1.10, 1.73)	1.36** (1.08, 1.72)	1.31 (0.99, 1.75)	1.67*** (1.23, 2.27)	1.58*** (1.15, 2.16)	1.37 (0.94, 2.01)
**V**	Mental and behavioural disorders	1.50*** (1.23, 1.84)	1.41*** (1.15, 1.73)	1.25* (1.02, 1.55)	1.79*** (1.30, 2.48)	1.58*** (1.15, 2.15)	1.07 (0.77, 1.47)
**VI**	Diseases of the nervous system	1.37*** (1.16, 1.63)	1.35*** (1.14, 1.60)	1.26** (1.07, 1.49)	1.65*** (1.32, 2.06)	1.62*** (1.30, 2.04)	1.35*** (1.10, 1.66)
**VII**	Diseases of the eye and adnexa	1.25*** (1.18, 1.33)	1.24*** (1.17, 1.32)	1.20*** (1.13, 1.27)	1.70*** (1.56, 1.87)	1.65*** (1.51, 1.81)	1.53*** (1.39, 1.69)
**VIII**	Diseases of the ear and mastoid process	1.56*** (1.33, 1.83)	1.54*** (1.31, 1.80)	1.47*** (1.27, 1.73)	2.00*** (1.53, 2.62)	1.99*** (1.51, 2.62)	1.92*** (1.41, 2.59)
**IX**	Diseases of the circulatory system	1.36*** (1.19, 1.56)	1.32*** (1.17, 1.49)	1.17*** (1.08, 1.26)	2.03*** (1.57, 2.62)	1.88*** (1.48, 2.40)	1.34*** (1.20, 1.49)
**X**	Diseases of the respiratory system	3.28*** (3.03, 3.55)	3.13*** (1.89, 3.40)	2.69*** (2.48, 2.93)	10.54*** (9.14, 12.16)	9.52*** (8.24, 11.01)	6.17*** (5.11, 7.44)
**XI**	Diseases of the digestive system	1.37*** (1.32, 1.42)	1.35*** (1.30, 1.40)	1.27*** (1.23, 1.32)	1.72*** (1.60, 1.86)	1.67*** (1.54, 1.80)	1.49*** (1.38, 1.62)
**XII**	Diseases of the skin and subcutaneous tissue	1.26*** (1.07, 1.47)	1.22** (1.06, 1.41)	1.10 (0.99, 1.23)	1.73*** (1.44, 2.07)	1.68*** (1.40, 2.02)	1.46*** (1.18, 1.81)
**XIII**	Diseases of the musculoskeletal system and connective tissue	1.44*** (1.37, 1.51)	1.42*** (1.35, 1.49)	1.30*** (1.23, 1.37)	2.01*** (1.81, 2.24)	1.93*** (1.74, 2.14)	1.67*** (1.49, 1.87)
**XIV**	Diseases of the genitourinary system	1.07 (0.85, 1.35)	1.06 (0.85, 1.31)	1.15 (0.99, 1.33)	1.68*** (1.19, 2.37)	1.50*** (1.14, 1.98)	1.13 (0.96, 1.33)

Jacobs A, *et al. BMJ Open Respir Res* 2023; 10:e002003. doi: 10.1136/bmjresp-2023-002003

**XV**	Pregnancy, childbirth and the puerperium	N/A	N/A	N/A	N/A	N/A	N/A
**XVI**	Certain conditions originating in the perinatal period	N/A	N/A	N/A	N/A	N/A	N/A
**XVII**	Congenital malformations and chromosomal abnormalities	1.27 (0.95, 1.71)	1.27 (0.95, 1.71)	1.19 (0.87, 1.62)	1.72 (0.91, 3.26)	1.50 (0.85, 2.67)	1.38 (0.77, 2.27)
**XVIII**	Non-classified symptoms and abnormal clinical findings	1.42*** (1.36, 1.48)	1.38*** (1.32, 1.44)	1.27*** (1.22, 1.32)	1.91*** (1.78, 2.04)	1.79*** (1.68, 1.92)	1.48*** (1.37, 1.59)
**XIX**	Injury, poisoning, consequences of external causes	1.38*** (1.31, 1.46)	1.34*** (1.27, 1.42)	1.25*** (1.19, 1.32)	1.82*** (1.66, 2.00)	1.72*** (1.56, 1.89)	1.48*** (1.34, 1.63)
**XX**	External causes of morbidity	N/A	N/A	N/A	N/A	N/A	N/A
**XXI**	Factors influencing health status and contact with health services	1.21*** (1.12, 1.32)	1.20*** (1.11, 1.31)	1.17*** (1.07, 1.28)	1.21*** (1.06, 1.38)	1.18** (1.04, 1.35)	1.05 (0.94, 1.18)
**XXII**	Codes for special purposes	N/A	N/A	N/A	N/A	N/A	N/A

Incident Rate Ratios (IRRs) are shown for each variable, derived from exponentiating regression coefficients. Confidence intervals (2.5%, 97.5%) are in brackets. Levels of model adjustment: minimally adjusted models were adjusted for the matching covariates (age, sex, ethnicity & location); intermediate adjusted models were adjusted for socioeconomic deprivation in addition to the matching covariates; fully adjusted models were adjusted for deprivation, smoking status, BMI category and comorbidities in addition to the matching covariates. Significance: *** $p<0.001$; ** $p<0.01$; * $p<0.05$.

References

1. British Thoracic Society. BTS/SIGN British guideline on the management of asthma [online] 2019 [67-68]. Available from: https://www.brit-thoracic.org.uk/document-library/guidelines/asthma/btssign-guideline-for-the-management-of-asthma-2019/ accessed September 5, 2022.
2. Zhang Q, Bush K, Nolan J, et al. Definitions of Asthma for UK Biobank Phase 1 Outcomes Adjudication [online]. *UK Biobank* 2018. https://biobank.ndph.ox.ac.uk/showcase/showcase/docs/alg_outcome_asthma.pdf (accessed August 6, 2021).
3. Shrine N, Portelli MA, John C, et al. Moderate-to-severe asthma in individuals of European ancestry: a genome-wide association study. *The Lancet Respiratory Medicine* 2019;7(1):20-34. doi: 10.1016/s2213-2600(18)30389-8
4. Ho D, Imai K, King G, et al. MatchIt: Nonparametric Preprocessing for Parametric Causal Inference. *Journal of Statistical Software* 2011;42(8):1 - 28. doi: 10.18637/jss.v042.i08
5. Greifer N. Covariate balance tables and plots: a guide to the cobalt package [online]. 2020; 10. https://cran.r-project.org/web/packages/cobalt/vignettes/cobalt.html (accessed September 12, 2021).
6. ICD-10-CM Official Guidelines for Coding and Reporting. Guidance for coding and reporting using the International Classification of Diseases, 10th Revision, Clinical Modification (ICD-10-CM): Centers for Medicare & Medicaid Services, 2023. Accessed June 15, 2023.
7. Zeileis A, Hothorn T. Diagnostic checking in regression relationships [online]. 2002 7-10; 2(3). https://journal.r-project.org/articles/RN-2002-018/RN-2002-018.pdf (accessed September 12, 2022).
8. Zeileis A, Köll S, Graham N. Various Versatile Variances: An Object-Oriented Implementation of Clustered Covariances in R. *Journal of Statistical Software* 2020;95(1):1 - 36. doi: 10.18637/jss.v095.i01
9. Zhou J, Wu R, Williams C, et al. Prediction Models for Individual-Level Healthcare Costs Associated with Cardiovascular Events in the UK. *Pharmacoeconomics* 2023;41(5):547-59. doi: 10.1007/s40273-022-01219-6
10. HRG4+ 2018/2019 reference costs grouper. https://digital.nhs.uk/services/national-casemix-office/downloads-groupers-and-tools/costing---hrg4-2018-19-reference-costs-grouper [program]: NHS Digital. Accessed June 1, 2021.
11. [dataset] National Schedule of NHS Costs 2018/19 https://www.england.nhs.uk/publication/2018-19-national-cost-collection-data-publication/: NHS England, 2021. Accessed 30 July, 2021.
